# Oral health care concerns among autism patients: A review

**DOI:** 10.6026/9732063002001017

**Published:** 2024-09-30

**Authors:** Sibani Sarangi, Rajeev Ranjan Raj, Anurag Rawat, Sajjad Salam, Shubham Tripathi, Dipanshu Aggarwal, Ritik Kashwani

**Affiliations:** 1 Department of Periodontology, Hitech Dental College and Hospital, Bhubaneswar, India; 2 Department of Psychiatry, District Hospital, Deoghar, Jharkhand, India; 3 Department of Cardiology, Himalayan Institute of Medical Science, Dehradun, India; 4 Department of Dental and Maxillofacial Services, Bahrain Defense Force Hospital, Bahrain; 5 Department of Conservative Dentistry and Endodontics, District Bhoj Hospital, Dhar, Indore, Madhya Pradesh, India; 6 Department of Oral and Maxillofacial Pathology and Oral Microbiology, Shree Bankey Bihari Dental College, Uttar Pradesh, India; 7 Department of Oral Medicine and Maxillofacial Radiology, School of Dental Sciences, Sharda University, Greater Noida, India

**Keywords:** Autism, harmonious oral environment, neural, orofacial dynamics, repetitive

## Abstract

Autism is a developmental disorder mostly affecting neural and developmental skills with problems in learning, communication and repetitive behaviour. It has become more highlighted over the past few decades because of the increasing awareness, survey tools and research worldwide. Existing worldwide, this disorder affects the normal lifestyle of the affected people since childhood and continues with the same pace through lifetime. Due to poor neuromuscular coordination, it also affects the normal orofacial dynamics of an individual and manifests as multiple oral disorders. Therefore it's of interest to report known data on the oral manifestations of this spectral disorder and various approaches at varied levels to maintain a harmonious oral environment.

## Background:

Autism, commonly known as "autism spectrum disorder" (ASD), is an array of complex, rapidly developing neuro-developmental disturbance manifesting as an impaired mental and emotional development [1]. Autopsying the term autism spectrum disorder, we find it to be a culmination of two words i.e "autism" and "spectrum" [2]. The term "autism" is derived from a Greek word "autos" meaning self which appropriately describes the characteristic feature of this disorder namely a profound withdrawal from people and from social reactions with people, even parents [3]. "Spectrum" because the vast fluctuations in the type and severity of disorder that patients experience [4]. So in a nutshell, autism can be discussed as a customized behavioural disorder that compels the patient to be gregarious [5]. In 1943, Leokanner after thorough research and studies put forth an inference about autism. It stated that autism becomes evident from a very tender age of 1 to 3 years and is therefore commonly known as Kanner syndrome. It is also famous as early infantile autism, infantile psychosis or childhood schizophrenia [6]. According to KOPEL (1977), a patient can be diagnosed with autism spectrum disorder if he/she confirms the following symptoms such as individuals with autism spectrum disorder (ASD) often exhibit a range of characteristic behaviors and challenges. Social withdrawal and a sense of extreme loneliness are common, along with language disturbances that may include mutism, parroting or echolalia and difficulty grasping the concept of "yes" or properly using personal pronouns. Many display an obsessive desire for sameness, reacting strongly to changes in routine and may also experience eating disturbances, such as holding food in their mouths or preferring a soft diet. There is often a pronounced fascination with spinning objects and engagement in repetitive, self-stimulatory behaviors. Additionally, hyperactivity, mental retardation, and nystagmus can accompany these behaviors, alongside an increased likelihood of seizure disorders. This complex array of symptoms requires tailored approaches to care and support [7]. Autism spectrum disorder is omnipresent in all racial, ethnic and socioeconomic groups. Found approximately in 5 out of 10,000 births, autism shows slight male predilection (4:1). However, this rate shows geographic and periodic variations [8]. Although the precise etiology of autism spectrum disorder (ASD) remains unclear, on-going research suggests that it is likely the result of a combination of genetic, environmental, prenatal, perinatal and neonatal risk factors. Studies indicate that multiple factors may contribute to the development of ASD, with genetic predispositions interacting with environmental influences and risks during critical periods of brain development. Understanding these complex interactions continues to be a focus of research aimed at uncovering the underlying causes of autism [9]. Recent studies exemplify that parameters such as CNTNAP2 gene, de novo mutations, highly maternally derived intrauterine androgen concentrations may be few tonics that boost up the pathophysiology of autism. Globally, more than 100 genomic cases have been detected in patients with ASD [10]. Several studies conducted in India have investigated the genetic basis of autism spectrum disorder (ASD) and identified potential genetic factors associated with the condition. Notable genes that have been implicated include the Engrailed 2 (EN2) gene, which plays a role in brain development; RELN, involved in neuronal migration; ITGB3, which affects cell adhesion; and SLC6A4, which is related to serotonin transport. Additionally, Monoamine Oxidase A (MAOA) on the X-chromosome has been associated with ASD. While these findings are significant, they are primarily derived from research conducted within India and necessitate further validation through global studies to confirm their relevance and applicability across diverse populations [11, 12]. Therefore, it is of interest to describe the concerns of oral hygiene in autistic patients.

## Methodology:

This article on the oral manifestations of autism spectrum disorder (ASD) and approaches to maintaining oral health is based on a thorough literature review and clinical observations.

The methodology includes data collection from databases like PubMed, Google Scholar, and Web of Science, focusing on studies from 2000 to 2023 that explore oral health in ASD. Both general and specific dental conditions in children and adults with ASD were analyzed, including dental caries, periodontal diseases, and oral habits. Geographic diversity in the studies ensured a well-rounded analysis. Clinical observations from dental practitioners treating ASD patients were integrated, offering practical insights into challenges like communication barriers and behavioural management during dental care. This provided a bridge between research and real-world application. The article also examines modern management strategies, such as speech therapy, behavioural interventions, and sensory integration therapy, in dental settings. Furthermore, recent technological advances, like virtual reality and the metaverse, were reviewed for their potential to desensitize ASD patients to clinical environments, improving patient care and outcomes during dental procedures.

## Perinatal, prenatal, and neonatal risk factors:

Perinatal, prenatal, and neonatal risk factors that may contribute to the development of autism spectrum disorder (ASD) include several important conditions and complications. Advanced maternal age has been associated with an increased risk of ASD. Fetal distress, which can occur due to various factors during pregnancy or labour, may also be a contributing factor. Gestational respiratory infections and labour complications can impact fetal development and increase the risk of ASD. Additionally, preterm birth, neonatal jaundice, delayed birth cry, and birth asphyxia are conditions that may trigger or exacerbate developmental issues linked to autism. These factors underscore the complexity of ASD's etiology, involving interactions between genetic predispositions and early-life challenges. There are few mere reasons that cannot be ruled out while discussing the etiology of autism. These include organic brain damage or dysfunction, inefficient environmental triggering of psychological factors, defective metabolic processes, personalities, attitudes and behaviour of parents [13, 14].

## Global prevalence and distribution of ASD:

On a global basis, autism has a heterogeneous distribution. No studies till date show similar results. The frequency and intensity of this spectrum disorder varies from place to place which may be acceptable owing to its geographic and racial diversity. One study reveals that autism has a frequency of 1:68 *i.e.* out of a study population of 68 patients, 1 patient confirms ASD. Approximately 1.4 out of 10,000 in Oman, 29 out of 10,000 in UAE, 4.3 out of 10,000 and 18 out of 10,000 in Saudi Arabia are diagnosed with autistic disorder spectrum [15]. According to the World health organization (WHO), 1 out of 160 children have autistic spectrum disorder. As already stated above, this ratio is also an average estimate and varies accordingly [16]. Though the study shows variable results, most of them are concentrated in the developed belt of the world excluding the developing and developed countries. Previously, this disorder was not much in the limelight as of now may be due to limited research ,lack of awareness ,constricted diagnostic criteria with mere diagnostic tools and most importantly the need to understand the need of reporting . However, things have gone for better over the past 50 years, where the reported cases number have escalated significantly making things lucid for both the patients as well as the care holders.

## Autism in the Indian context:

Indian Scenario of Autism is that overall rate of diagnosed autism disorders have increased over years on the global scale, the rate of diagnosis has also increased on a national level. The summative inference of multiple researches conducted in India over the past few years gives us an overall incidence of 0.15% to 1.01% of the study population being affected with ASD. These results were mostly collected using variable study methods and diverse areas. According to a study, INCLEN, multiple study groups were designed according to age and geographic areas [17]. The result showed as such that the prevalence of autism spectrum disorder (ASD) varies by age group and geographical area. For different age groups, the prevalence is approximately 1 in 125 for children aged 3-6 years and 1 in 85 for those aged 6-9 years. Geographically, the prevalence rates differ: rural areas have about 0.9% of ASD cases, urban areas have 1.01%, hill areas have 0.6%, tribal areas have 0.1%, and coastal areas have 0.61%. It is important to note that these figures are based on existing research and the cases identified during data collection. The prevalence rates may vary over time as research progresses and diagnostic tools improve, which could lead to more accurate and comprehensive data on ASD [17].

## Oral health in patients with ASD:

The conclusion about oral manifestations in an autistic patient is quite more fluctuating than its etiology and frequency. Contrasting outcomes have been put forth about the oral health status of autistic spectrum disorder patients owing to its narrow range of study and research. Despite deficient oral hygiene practices, there is no such specific oral disorder that can be marked as the clinical diagnostic signature of this disorder [18]. Some researchers have proved high incidence of caries and periodontal diseases in autistic patients while few researches defies the former. With a cut shot competition between the two inferences, the final verdict shows slight predilection towards poor oral hygiene conditions being more prevalent among patients with autism spectrum disorder. Patients with autism spectrum disorder (ASD) often encounter a range of dental problems, including dental caries, periodontal issues, deleterious oral habits, and tooth eruption disorders. They are more susceptible to cavities due to challenges in maintaining oral hygiene and dietary habits [19]. Periodontal problems, such as gingivitis and periodontitis, are common, often resulting from inadequate oral care. Additionally, many ASD patients exhibit harmful oral habits, such as thumb sucking or lip biting, which can adversely affect their dental health. Tooth eruption disorders are also prevalent, with delays or abnormalities in the eruption of teeth impacting overall dental development and alignment. These issues highlight the need for tailored dental care strategies to address the specific needs of ASD patients [20, 21]. Though autism and dental caries are two independent entities, yet are quite relatable sometimes because of the latter being quite common among the former patients. Few studies held over different parts of the world gives this condition a strong confirmation. In 2011, a study conducted in Dubai on a population size of 61 patients showed a caries incidence in 47 out of 61 patients constituting approximately 77% of study size. Another study in Chennai on a population size of 483 showed approximately 24% of the population to have a carious tooth. This status can be attributed to the affinity of autistic spectrum disorder patients towards soft and sweetened foods. Further, these patients have a tendency to pouch the food in their mouth for an extended period of time. The culmination of these two behavioural patterns worsens the condition leading to an escalated risk of caries. Though a major factor behind such oral conditions in patients with ASD disorder, these are not the only factor behind the onset of carious teeth in autistic patients. Some factors have miniscule input in the onset and sequelae of dental caries. Such few factors are intake of psychoactive drugs or anticonvulsants that cause xerostomia and hence, caries [22, 23]. Autism spectrum disorder patients have limited manual dexterity and poor muscle tone for which they end up having irregular brushing techniques. This causes sustained piling up of debris and sugar laden food in the periodontal structures leading to breakdown of periodontal framework that manifests as gingivitis (both localized and generalized), periodontitis, attachment loss *etc.* Few studies conducted among autistic population both at global levels and in India shows an average of 50% or more of cases showing signs of compromised periodontal status that manifests mostly as mild gingivitis with a thin film of visible plaque that covers more than one than one third of tooth surface and is visible through naked eye [24, 25](Table 1).

## Oral habits in ASD patients:

Oral Habits:

Despite poor oral hygiene and high caries risk, autistic patients very often practise deleterious habits. Some commonly followed practices are drooling, tongue thrusting and dysphagia. The tendency of this habit is more because of poor muscle tone than of psychological stress. According to a study conducted in an age group of 20-41 years on a population size of 30, approximately 18 out of 30 (60%) had bruxism. This habit often causes regressive alteration of the tooth with saucer shaped coronal features often evident as loss of enamel structure [26]. Despite the commonly encountered oral problems in autistic spectrum disorder patients, one should not completely rule out the incidences of self-inflicted injuries, repetitive bruises, cuts, trauma, crowding, open bite, ogival palate, fistula, gingival hyperplasia and oral ulcerations. Despite an unavoidable habit, the above-mentioned oral habits, self-inflicted injuries and orthodontic problems should be looked upon with utmost care and suspicion. Tooth Eruption Disorders: Delayed tooth eruption may also be observed in some patients with autistic features because of these patients being mostly under phenytoin therapy [27].

## Comprehensive management of autism spectrum disorder (ASD):

Managing a patient with autism spectrum disorder (ASD) requires a multi-faceted and collaborative approach involving patience and structured strategies. The primary components of effective management include speech therapy, behaviour treatments, sensory integration therapy and medications to address co-occurring conditions. A structured approach enhances the effectiveness of these interventions, improving outcomes for individuals with ASD [28]. Speech therapy helps ASD patients develop communication skills, enabling them to convey their needs and thoughts, which fosters positive relationships with caregivers and peers [29]. Behaviour management techniques address challenging behaviors while promoting positive ones. These techniques include non-pharmacological methods such as audioanalgesia, biofeedback, hypnodontics and relaxation strategies. These interventions aim to reduce anxiety, improve behaviour, and enhance the therapeutic experience [30]. Sensory integration therapy aids patients in processing sensory input, helping them distinguish between colours, sounds and sensations. It improves their ability to manage sensory stimuli and reduces sensory overload [31]. Medications, such as conscious sedation and behaviour-shaping drugs, are used to manage symptoms and improve functioning in ASD patients. Conscious sedation (*e.g.* nitrous oxide) is helpful during dental procedures, though it must be administered cautiously due to its potential side effects on DNA synthesis. General anesthesia is reserved for more invasive procedures or when the patient cannot cooperate. Behaviour-shaping medications address aggression and hyperactivity and when combined with therapies, improve the patient's overall well-being [32]. Maintaining oral hygiene in ASD patients can be challenging due to their unique needs. Building trust and establishing routines are crucial. Selecting preferred toothbrushes and toothpaste and using high-fluoride products (1400-1500 ppm) can help improve oral hygiene. Fluoride mouth rinses with no alcohol content are recommended to prevent irritation [33, 34]. In dental offices, understanding the patient's psychological state is essential for tailoring communication and treatment. Patience, gentle approaches, consistency, and behavioural management techniques such as the tell-show-do method foster cooperation. In some cases, sedation or immobilization devices may be necessary for uncooperative patients. Positive reinforcement helps encourage cooperation [35]. Recent advances, such as the metaverse, offer promising tools for ASD care. Immersive virtual environments can help desensitize patients to real-world stressors, allowing doctors to simulate situations like dental visits or social interactions. The metaverse also enables doctors to train in managing ASD patients and collaborate with professionals worldwide, improving treatment protocols [36].

## Conclusion:

Autism spectrum disorder (ASD), though has merged slowly over years and is now quiet common as compared to its past record, it still remains a mystery that needs to be further unboxed. It never comes with a manual, rather it comes with a parent that never gives up. Management and care of these patients is quite difficult but can be dealt with effectively with utmost planning and patience. However, before proceeding to the management aspect, its diagnosis at the correct age is of much more significance because the longer a child with autism goes without help the harder they are to reach.

## Figures and Tables

**Table 1:** Risk factors, prevalence, and oral health challenges in patients with autism spectrum disorder (ASD)

**Category**	**Details**
Risk Factors for ASD [13,14].	Advanced maternal age, fetal distress, gestational respiratory infections, labour complications, preterm birth, neonatal jaundice, delayed birth cry, birth asphyxia, organic brain damage, environmental triggers, metabolic dysfunctions, parental behaviour
Global Prevalence of ASD [16].	Varies widely by location: Oman (1.4/10,000), UAE (29/10,000), Saudi Arabia (4.3/10,000 and 18/10,000) [15]; WHO estimate: 1 in 160 children globally.
Indian Prevalence of ASD [17]	Varies by age and region: 1 in 125 (ages 3-6), 1 in 85 (ages 6-9). Rural areas (~0.9%), urban areas (~1.01%), hill areas (~0.6%), tribal areas (~0.1%), coastal areas (~0.61%).
Oral Manifestations of ASD [18-21].	No specific oral disorder is consistently associated with ASD; however, poor oral hygiene, dental caries, periodontal disease, and deleterious oral habits are common.
Dental Caries in ASD Patients [22,23]	Study in Dubai (2011): 77% of 61 patients with ASD had caries. Study in Chennai: 24% of 483 patients with ASD had caries. Soft and sweetened foods, food pouching behaviour, psychoactive drugs, and anticonvulsants contribute to caries risk.
Periodontal Health in ASD Patients [24,25]	Limited manual dexterity and poor muscle tone lead to irregular brushing, plaque buildup, and compromised periodontal health, with gingivitis and periodontitis commonly observed.
Oral Habits in ASD Patients [26]	Common habits include drooling, tongue thrusting, dysphagia, bruxism (60% in a study of 30 patients). These habits lead to enamel loss, self-inflicted injuries, crowding, open bite, and other orthodontic problems.
Tooth Eruption Disorders in ASD [27]	Delayed tooth eruption, often linked to phenytoin therapy in ASD patients.

## References

[1] Faras H (2010). Ann Saudi Med..

[2] Delli K (2013). Med Oral Patol Oral Cir Bucal..

[3] Orellana LM (2012). Med Oral Patol Oral Cir Bucal..

[4] Jaber MA (2011). J Appl Oral Sci..

[5] Vishnu Rekha C (2012). Eur Arch Paediatr Dent..

[6] Harris J (2018). Int Rev Psychiatry..

[7] Kopel HM (1977). ASDC J Dent Child..

[8] Hirota T, king HB (2023). JAMA..

[9] Nelson SB, Valakh V (2015). Neuron..

[10] Novara F (2010). Clin Genet..

[11] Young LEA (2019). J Neurogenet..

[12] Pal AK (2018). J Hum Reprod Sci..

[13] Kim YS (2011). Am J Psychiatry..

[14] Hogart A (2010). Neurobiol Dis..

[15] Baio J (2018). MMWR Surveill Summ..

[16] Celep F (2006). Eur J Obstet Gynecol Reprod Biol..

[17] Orellana LM (2012). Med Oral Patol Oral Cir Bucal..

[18] El Khatib AA (2014). Int J Paediatr Dent..

[19] Du RY (2015). Autism..

[20] Hippler K, Klicpera C (2003). Philos Trans R Soc Lond B Biol Sci..

[21] Hyman SL (2020). Pediatrics..

[22] Centers for Disease Control and Prevention. (2007). MMWR Surveill Summ..

[23] Bhandary S, Hari N (2017). Eur Arch Paediatr Dent..

[24] Arora A (2011). J Investig Clin Dent..

[25] Shetty S (2010). J Indian Prosthodont Soc..

[26] Stelly K (1974). Fogorv Sz..

[27] Rama L (2023). Rev Med Suisse..

[28] Greenwell T (2021). Am J Speech Lang Pathol..

[29] Hart R (2010). Emotional Behav Diff..

[30] Hoehn TP, Baumeister AA (1994). J Learn Disabil..

[31] Wahr JA (2017). Br J Anaesth..

[32] Kogan MD (2009). Pediatrics..

[33] Centers for Disease Control and Prevention. (2012). MMWR Surveill Summ..

[34] Kashwani R, Sawhney R (2023). Int Dent J Stud Res..

[35] Kashwani R (2024). Comm Pract..

[36] Kwon TH (2021). Int Dent J..

